# A Plan for Community Event-Based Surveillance to Reduce Ebola Transmission — Sierra Leone, 2014–2015

**Published:** 2015-01-30

**Authors:** Sam Crowe, Darren Hertz, Matt Maenner, Ruwan Ratnayake, Pieter Baker, R. Ryan Lash, John Klena, Seung Hee Lee-Kwan, Candice Williams, Gabriel T. Jonnie, Yelena Gorina, Alicia Anderson, Gbessay Saffa, Dana Carr, Jude Tuma, Laura Miller, Alhajie Turay, Ermias Belay

**Affiliations:** 1 Epidemic Intelligence Service, CDC; 2 CDC Sierra Leone Ebola Response Team; 3 International Rescue Committee; 4 Bo District Health Management Team, Sierra Leone Ministry of Health and Sanitation; 5 World Health Organization

Ebola virus disease (Ebola) was first detected in Sierra Leone in May 2014 and was likely introduced into the eastern part of the country from Guinea (1). The disease spread westward, eventually affecting Freetown, Sierra Leone’s densely populated capital. By December 2014, Sierra Leone had more Ebola cases than Guinea and Liberia, the other two West African countries that have experienced widespread transmission (2). As the epidemic intensified through the summer and fall, an increasing number of infected persons were not being detected by the county’s surveillance system until they had died (Figures 1 and 2). Instead of being found early in the disease course and quickly isolated, these persons remained in their communities throughout their illness, likely spreading the disease.

In October 2014, members of the International Rescue Committee (IRC), Sierra Leone’s Bo District Health Management Team (DHMT), and CDC developed the Community Event-Based Surveillance (CEBS) system* to help strengthen the country’s Ebola surveillance and response capabilities. It consists of community health monitors who are trained to detect trigger events (Box) thought to be associated with Ebola transmission to find possible cases early in the course of disease, and surveillance supervisors who investigate reported events and isolate and begin treating persons with suspected Ebola.† It is not intended to replace the current system, but to supplement case-finding and contact tracing, the core of Ebola surveillance in the West African response (5,6). CEBS is being pilot tested in Sierra Leone’s Bo District and recently has been adopted as part of Sierra Leone’s national surveillance strategy in low- and medium-transmission districts.§ It will be deployed to other parts of the country soon. This report describes the CEBS system, plans for its evaluation, and some expected benefits and challenges.

## Pilot Overview

In November 2014, the IRC implementation team chose two chiefdoms (Gbo and Selenga) in Bo District as pilot areas to assess the feasibility and acceptability of CEBS.¶ Local community health officers, who serve as clinical staff and health care facility administrators, consulted with the Gbo and Selenga paramount chiefs and chose community health monitors (e.g., teachers, farmers, or other community members who are knowledgeable about their village and its inhabitants) from participating villages.

Monitors are trained to detect and to report trigger events selected by the Bo District community health officers that might indicate introduction or presence of Ebola in a village, such as signs of illness among family members, friends, health care workers, funeral attendees, or travelers. Monitors function alongside the district contact tracers, but focus on detecting trigger events, which might involve previously unknown contacts. Monitors are provided with cellphones in a closed user group to facilitate communication, and receive a stipend to compensate for time spent away from their regular work.

When a monitor learns of a trigger event in the village, he or she reports the event to a local community surveillance supervisor. Supervisors are responsible for investigating trigger events and determining whether these indicate suspected Ebola cases. The supervisor must visit the affected village and conduct the investigation within 24 hours of the initial call. To ensure timely and consistent reporting, supervisors call monitors every week to check for missed alerts and to confirm that the monitors did not detect any Ebola trigger events. The supervisors document all calls with monitors, including those that do not result in detection of a suspected case.

If, after reviewing the monitor’s notification and conducting an investigation, the supervisor suspects that there might be an Ebola case in a village, the supervisor contacts the local community health officer for guidance. Community health officers might visit affected villages to assist the monitor and supervisor in complicated or sensitive situations. When a supervisor finds a suspected Ebola case, he or she isolates the person at the periphery of the village, notifies the district Ebola surveillance office, and requests transportation for that person to a holding center, where staff collect blood specimens for Ebola virus testing. The supervisors carry sachets of oral rehydration salts to initiate early treatment, and packets of powdered bleach (with instructions for use) to provide to households with suspected cases to disinfect surfaces possibly contaminated with infected body fluids. With the assistance of the patient, the supervisor creates a line list of contacts and provides it to the district contact tracing team for follow-up.

BOX **Ebola trigger events for community health monitors — Community Event-Based Surveillance system, Sierra Leone, 2014–2015**

**Table:** 

Two or more ill or dead family members, household members, or friends. One ill or dead traveler in the village (the traveler could be someone from the village who left and returned or someone who is not from the village). One ill or dead health care worker in the village. One ill or dead person who was a contact of a suspected Ebola case and was not known to be tracked by a contact tracing team. One ill or dead person who attended a funeral within the preceding 3 weeks. Any traditional burial that took place in the village or surrounding community (this event trigger will not generate a suspected case investigation, but will alert the surveillance and response team that there might be multiple cases in the near future).

## Evaluation Plan

Preliminary assessments in December 2014 indicated that the pilot implementation in Bo District has had a high level of acceptance by key community leaders, villagers, and the case detection and response team members. Plans are being developed to expand CEBS to other chiefdoms in Bo District and other districts in Sierra Leone in the near future, making it possible to conduct an evaluation of its effectiveness in different parts of the country. The evaluation will include an assessment of the following system attributes: 1) the sensitivity and specificity of case detection (the number of cases detected by CEBS that were not found by contact tracers and did not generate alerts through the existing system, and the proportion of actual alerts); 2) the positive predictive value of the trigger events (the proportion of suspected cases detected by each trigger event that are confirmed to be actual cases); 3) the timeliness of reporting and response (the mean and median number of days from illness onset to specimen collection among detected cases before and after implementation of the system); and 4) the acceptability of the system, based on interviews of key informants in a sample of villages.

## Expected Benefits and Challenges

Prompt detection and isolation of persons with Ebola is expected to lead to a number of key public health benefits. First, immediate isolation of infected persons and provision of bleach to affected households should reduce household contact with infectious body fluids and thereby limit disease spread (7). Second, decreasing the number of persons who die from Ebola in the community will also decrease the occurrence of burials by relatives, friends, and neighbors, which can address another route of Ebola virus transmission (8). Third, conducting investigations within 24 hours of case detection should help find patients at an earlier stage of illness and result in their arriving at an Ebola treatment unit much sooner. Fourth, initiating early oral rehydration therapy should help reduce dehydration, and might improve clinical outcomes (9). Fifth, training local Sierra Leoneans to monitor their villages for signs of disease spread can create a community-level surveillance infrastructure that can be used even after the epidemic in West Africa ends. This infrastructure, if established throughout the country, could help detect residual Ebola transmission and future Ebola outbreaks, and could even be used for other infectious diseases (3). In addition to these benefits, the system would likely increase community involvement and participation in the Ebola response, resulting in ownership of Ebola prevention activities and enhanced acceptance of key prevention messages.

Despite these benefits, challenges associated with implementation of CEBS will include recruiting and training staff, maintaining the communication and response network, monitoring participating villages for any concerns with CEBS operations, ensuring adequate transportation for the anticipated increased number of patients to the holding centers, and working with the holding centers to manage the expected increase in false-positive suspected cases. The implementation team will be monitoring these and other challenges throughout the pilot and as the system is expanded into other areas.

## Figures and Tables

**FIGURE 1:**
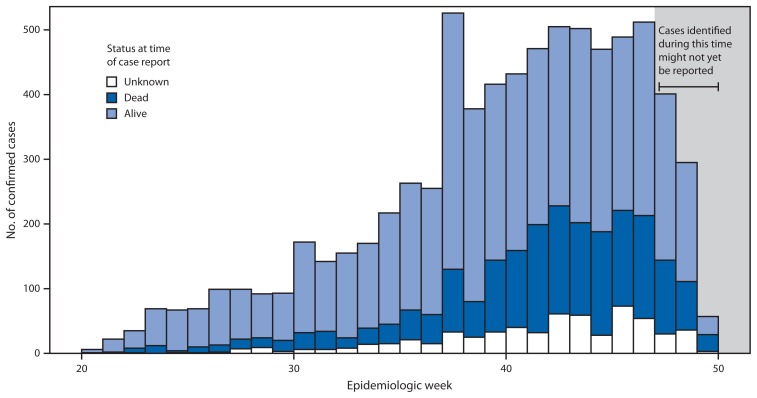
Number of confirmed cases of Ebola virus disease, by epidemiologic week and status at time of case report — Sierra Leone, May–December 2014 **Source:** Sierra Leone’s Epi Info Viral Hemorrhagic Fever database.

**FIGURE 2:**
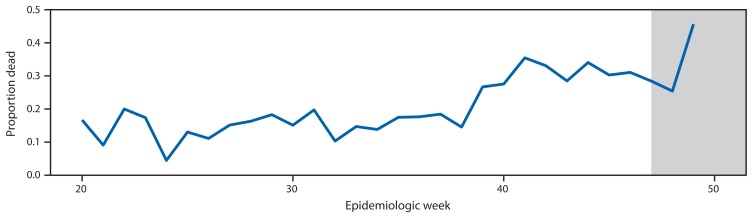
Proportion of persons with confirmed cases of Ebola virus disease who were already dead at time of case report, by epidemiologic week — Sierra Leone, May–December 2014 **Source:** Sierra Leone’s Epi Info Viral Hemorrhagic Fever database.

## Footnotes

* This type of surveillance has been used in previous Ebola outbreaks in Uganda (3,4) in addition to outbreaks of other infectious diseases, such as polio and influenza, throughout the world.

† In Sierra Leone, there are three ways to meet the suspected case definition: 1) a person must have a temperature >100.4°F (>38.0°C) and three or more symptoms associated with Ebola, such as vomiting, diarrhea, abdominal pain, headache, joint pain, fatigue, or unusual bleeding; 2) a person must have a fever and have been in contact with a confirmed case in the preceding 3 weeks; or 3) a person must be bleeding for an unexplained reason.

§ Sierra Leone has 14 districts, which comprise 149 chiefdoms. Each chiefdom is further divided into sections and then into villages. Bo District consists of 15 chiefdoms and approximately 1,000 villages and has both rural and urban areas. The second largest city in the country, Bo Town, is located in Bo District. Bo District has one Ebola holding center, one Ebola treatment unit, and a CDC laboratory that tests for Ebola.

¶ There are 43 villages in Gbo and 32 in Selenga, with a combined estimated population in the two chiefdoms of approximately 13,000. All 75 villages in Gbo and Selenga are participating in the pilot.
